# Bird evolution: testing the Metaves clade with six new mitochondrial genomes

**DOI:** 10.1186/1471-2148-8-20

**Published:** 2008-01-23

**Authors:** Mary Morgan-Richards, Steve A Trewick, Anna Bartosch-Härlid, Olga Kardailsky, Matthew J Phillips, Patricia A McLenachan, David Penny

**Affiliations:** 1 Allan Wilson Center for Molecular Ecology and Evolution, Massey University, Palmerston North, New Zealand; 2 Ecology Group, INR, Massey University, Palmerston North, New Zealand; 3 Department of Genetics, Cell and Organism Biology, University of Lund, Lund, Sweden; 4 Centre for Macroevolution and Macroecology, School of Botany and Zoology, Australian National University, Canberra, Australia

## Abstract

**Background:**

Evolutionary biologists are often misled by convergence of morphology and this has been common in the study of bird evolution. However, the use of molecular data sets have their own problems and phylogenies based on short DNA sequences have the potential to mislead us too. The relationships among clades and timing of the evolution of modern birds (Neoaves) has not yet been well resolved. Evidence of convergence of morphology remain controversial. With six new bird mitochondrial genomes (hummingbird, swift, kagu, rail, flamingo and grebe) we test the proposed Metaves/Coronaves division within Neoaves and the parallel radiations in this primary avian clade.

**Results:**

Our mitochondrial trees did not return the Metaves clade that had been proposed based on one nuclear intron sequence. We suggest that the high number of indels within the seventh intron of the β-fibrinogen gene at this phylogenetic level, which left a dataset with not a single site across the alignment shared by all taxa, resulted in artifacts during analysis. With respect to the overall avian tree, we find the flamingo and grebe are sister taxa and basal to the shorebirds (Charadriiformes). Using a novel site-stripping technique for noise-reduction we found this relationship to be stable. The hummingbird/swift clade is outside the large and very diverse group of raptors, shore and sea birds. Unexpectedly the kagu is not closely related to the rail in our analysis, but because neither the kagu nor the rail have close affinity to any taxa within this dataset of 41 birds, their placement is not yet resolved.

**Conclusion:**

Our phylogenetic hypothesis based on 41 avian mitochondrial genomes (13,229 bp) rejects monophyly of seven Metaves species and we therefore conclude that the members of Metaves do not share a common evolutionary history within the Neoaves.

## Background

The study of avian phylogeny abounds with examples of both unstable taxonomy and apparent convergent evolution. Indeed, in many cases the incidence of these conditions is correlated because taxonomists are at times misled by convergence in their systematic inferences. Progress with resolving avian phylogeny and thus identification of morphological/behavioral convergence has been enhanced by the use of molecular data that provides a source of characters independent of morphology. In particular, the use of large scale DNA, and in particular mitochondrial, sequence data has proved beneficial in stabilizing the avian tree [1,2].

**Figure 1:**
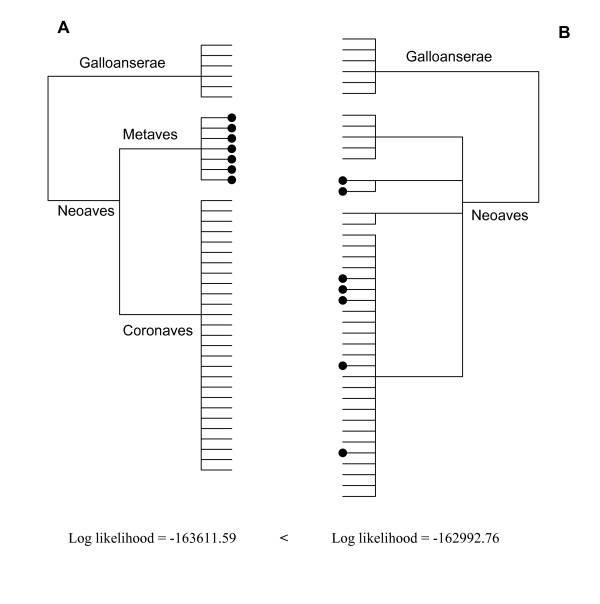
**The null hypotheses of modern bird (Neoaves) relationships**. (A) Metaves monophyly (based on Fain and Houde's 7^th ^intron of the β-fibrinogen tree); (B) Paraphyly of Metaves and Coronaves (based on Cracraft 1981). The likelihood scores of these trees were compared using our complete mitochondrial genome data set and tree (B) was better by 618 log-likelihood units, (P < 0.0001; SH test).

In contrast, a very different perspective on the phylogeny of birds suggesting a very different biogeographic history and classification was recently inferred from analysis of a single nuclear locus. Fain and Houde [3] proposed a major new division of Neoaves (that is, all extant birds except paleognaths [ratites and tinamous] and Galloanserae [ducks, chicken and relatives], see Figure 1). Their division of Neoaves into Metaves and Coronaves was based on data from the seventh intron of the β-fibrinogen gene (FGB-int7), including insertions and deletions. The proposed new group of Metaves comprises an intriguing and eclectic set of taxa, including the swifts, hummingbirds, flamingos, tropicbirds, grebes and kagu. Fain and Houde [3] liken the division of Neoaves into two major clades to the well known convergence of marsupial and placental mammals and list eleven examples of ecological and/or morphological convergence among Metaves and Coronaves. Their examples of convergence are in most cases, widely accepted and include for example the convergence of form and feeding of swallows and swifts as aerial insectivores. However, in contrast to the placental/marsupial division that is supported by at least 50 anatomical and physiological synapomorphies [4-6] the Metaves clade does not have a single published morphological character to support it, and there is no clear geographic separation from Coronaves.

Although the proposed Metaves/Coronaves division is supported by analysis of FGB-int7, there has, as yet been no similar pattern obtained from other genes. For example, Ericson et al. [7] confirm that although they also found the Metaves/Coronaves split with the FGB-int7 data, it was not evident in analyses of four other nuclear loci (and Chubb [8] has a fifth nuclear locus). Curiously though, when the four nuclear loci were concatenated with FGB-int7 for phylogenetic analysis, this increased the support for the Metaves/Coronaves split compared with FGB-int7 by itself [7]. In a cladistic analysis of morphological data Livezey and Zusi [9] did not find support for Metaves and declared it to be a 'nomen nudum' (name published without an adequate description). Thus, there is just a single nuclear locus giving a signal for Metaves, and it is therefore essential to test the proposed division using other classes of data. We examine here support for the Metaves/Coronaves split using data from complete mitochondrial (mt) genomes.

The avian tree from complete mitochondrial genome DNA sequence is expanding steadily [1,2,10] and is in agreement with morphological and nuclear DNA data in rooting birds between paleognaths and neognaths [2]. Mitochondrial introgression does not misled phylogenetic inferences at the depth of the divergence of the avian radiation and the range of mutation rates among mitochondrial genes make this an excellent single loci to study. However, previous mitochondrial datasets could not test the Metaves/Coronaves hypothesis. For example, Gibb et al. [1] and Slack et al. [2] did not include any members of the Metaves, and Watanabe et al. [10] and Yamamoto et al. (unpublished) report one each; great crested grebe and tropicbird respectively. Fortunately, we now have the complete sequence of the mitochondrial genome from seven bird species classified as Metaves by Fain and Houde [3]. The five reported here are the ruby-throated hummingbird (*Archilocus colubris*), the common swift (*Apus apus*), Australian little grebe (*Tachybaptus novaehollandiae*), kagu (*Rhynochetos jubatus*) and flamingo (*Phoenicoptera ruber roseus*). To these five we add two Metaves mt genomes from Genbank; great crested grebe (*Podiceps cristatus*) and tropicbird (*Phaethon rubricauda*), reported by Watanabe et al. [10] and Yamamoto et al. (unpublished, AP009043) respectively. In addition, we report a New Zealand rail (takahe, *Porphyrio hochstetteri*) because it allows us to distinguish between the traditional view of kagu and rails as being members of the same order (Gruiformes [11,12]) and the novel view of kagu in Metaves and rails in Coronaves [3].

In the process of testing the Metaves/Coronaves division, the mitochondrial genomes of the new taxa reported allow us to appraise other hypotheses about the phylogeny of modern birds. Irrespective of the validity of the Metaves/Coronaves division, we expect the hummingbird and swift to be sister taxa in this dataset (Apodiformes [11,9]). The position of the Gruiformes (including rails and kagu) is more equivocal; Livezey and Zusi [9] placed the rails very deep in the Neoavian tree and sister to the shorebirds (Charadriiformes) but Cracraft [11,13] infered the rails (and the kagu) as much more recently derived than the earliest Neoaves, within a large group comprising the raptors, shore, sea and aquatic birds.

Of the more controversial examples of avian ecological convergence proposed we can now test with analysis of complete mitochondrial genomes whether grebes and loons have converged on their form as "foot-propelled divers with high wing loading" [3,14]. Several authors have suggested that loons, flamingos, and grebes belong in the same part of the avian tree [15] and this grouping can also be tested. A consensus on the placement of flamingos has not been achieved [9,12,16] although many now consider the flamingos to be close relatives of grebes [8,14,17,18]. The name Mirandornithes was coined by Sangster [19] for the flamingo/grebe clade and this relationship is supported by a shared derived louse fauna [20]. However, recent analysis of morphological data found grebes and loons to be sister taxa, with neither being closely related to flamingo [9]. The sister group to the putative flamingo/grebe clade remains unresolved; morphology indicates flamingos are sister to the shorebirds (Charadriformes [16]) or have a highly aquatic ancestor [18], whilst DNA-DNA hybridization indicates tropicbirds are closest [14]. Within the Metaves, Fain and Houde [3] placed grebes closer to kagu and hummingbird/swift than to flamingo, although resolution within Metaves is generally poor.

The relative positions of the tropicbird and frigatebird are also uncertain. Traditionally they have been considered as part of the Pelecaniformes (pelicans, shags, darters, gannets, boobies, frigatebirds and tropicbirds) but this is now in question [21,9]. Tropicbirds may be in the Metaves clade [3], or basal to the vultures and penguins [22] and pelicans might be closer to the shoebill and hamerkop than to booby, darter and cormorant [14]. Putative Pelecaniformes are represented in our data set by the pelican, tropicbird and frigatebird.

### Seventh intron of β-fibrinogen

To return to the data supporting the Metaves/Coronaves clades, one source of ambiguity or false signal with FGB-int7 is the nucleotide sequence alignment. The FGB-int7 dataset on 147 bird species before alignment consists of DNA sequences that vary in length from 620 bp to 1155 bp but, when aligned, the dataset stretches to 1930 nucleotide sites. Furthermore, no single site is conserved across all taxa and indels are so frequent in this intron that every site is coded as a gap in at least one bird. Without conserved sites, alignment of the DNA sequences is problematical at best, and could lead to false phylogenetic signal [23]. Erroneous alignment can result in apparent phylogenetic signal among non-homologous sites (or gaps) and thus mislead tree reconstruction [24].

The conservative approach to alignment that we use with mitochondrial protein sequence is to remove all gaps and flanking sites on either side of the gap back to a constant site. If this alignment approach was applied to the FGB-int7 dataset of 147 birds there would be no sites left in the alignment! On the other hand, if an alignment could be confirmed, then the presence/absence of major features (such as major gaps) could be informative as 'rare genomic changes' [25]. A good example is the case of retroposed elements identified in placental evolution by Kriegs et al. [26]. In general, we know that when there are a very large number of character states, parsimony is a maximum likelihood estimator [27]. Thus gaps in DNA sequences, if they are uniquely definable, are expected to be excellent characters for phylogeny and have, for example, been useful in resolving parrot evolution [28]. The major question with the FGB-int7 data therefore remains whether the sequences can be reliably aligned.

Another potential difficulty with the Metaves/Coronaves result which is based on a single nuclear locus, is that many nuclear genes exist as multiple (paralogous) copies [29]. It is possible that, by chance, one paralogue was amplified and sequenced in some species (Metaves) and an alternative copy in other species, thus resulting in two clades.

We use four approaches to help evaluate the FGB-int7 sequences for phylogenetic reliability. We located the parsimony sites that unite Metaves. We examined the sensitivity of the final alignment to the reference tree that guides the DNA sequence alignment in programs such as Clustal X, to detect whether an alternative tree would give an equally good alternative alignment. We then reversed the direction of the sequences for alignment which is a proven method for detecting cases where alignment is relatively arbitrary [30]. Finally, we tested whether there was any evidence of paralogous copies by amplifying, cloning and sequencing FGB-int7 from two bird species.

## Results

### Complete mitochondrial genomes

The six new mitochondrial genome sequences have been deposited in GenBank under the following accession numbers: ruby-throated hummingbird (*Archilocus colubris*: EF532935, > 16,356 bp [incomplete due to repeats in the control region]), common swift (*Apus apus*: AM237310 EMBL, 17,037 bp), Australian little grebe (*Tachybaptus novaehollandiae*: EF532936, 18,002 bp), kagu (*Rhynochetos jubatus*: EF532933, 16,937 bp)) and greater flamingo (*Phoenicoptera ruber roseus*: EF532932, 17,446 bp); a New Zealand rail (takahe, *Porphyrio hochstetteri*: EF532934, 16,988 bp). The standard avian gene order was found in five of these birds, the exception being the flamingo which has the remnant of a second control region as seen in the falcon (CR2 [1,31]). Control region sequence was not used in the analysis.

### Phylogenetics

**Figure 2:**
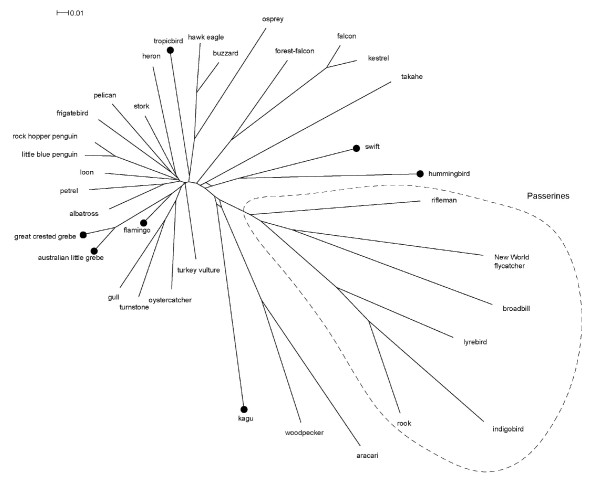
**Evolutionary relationships of modern birds based on complete mitochondrial genomes**. Unrooted consensus network of Neoaves based on Bayesian analysis of 35 complete avian mitochondrial DNA sequences. The consensus network includes all splits returned by > 25% of trees. Members of Metaves (Fain and Houde 2004) are indicated with black spots and (for convenience) passerines are indicated by an oval.

**Figure 3:**
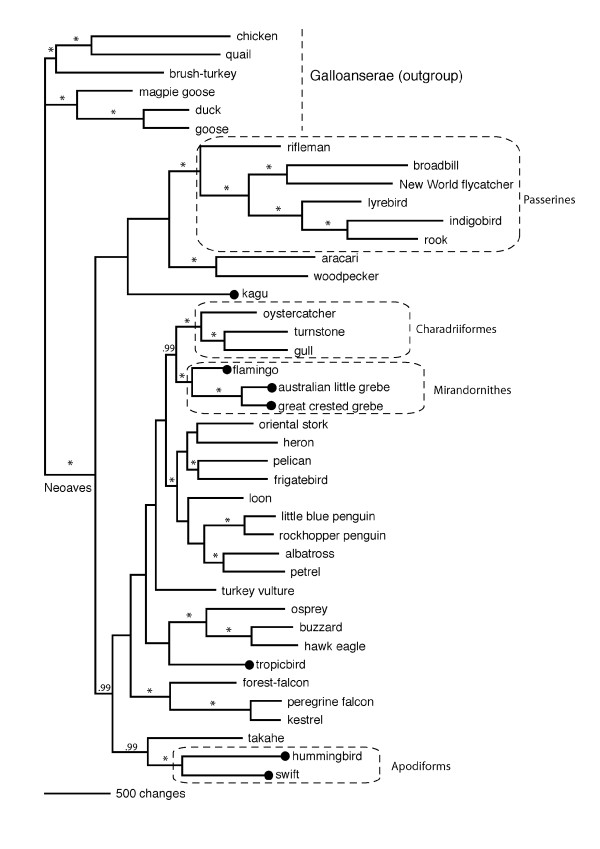
**Rooted tree of modern birds based on complete mitochondrial genomes**. Evolutionary relationships of Neoaves based on Maximum Likelihood analysis of 41 complete mtDNA sequences using a Galloanserae outgroup. Members of Metaves (Fain and Houde 2004) are indicated with black spots. Bayesian probabilities of 1.0 are indicated on branches as (*), branches with probabilities less than 0.99 not marked.

We have previously found with both birds [32] and mammals [33], as well as with simulated data [34,35], that the addition of an outgroup can disrupt a well-established ingroup tree, typically due to long-branch attraction. The ingroup tree without outgroups is more likely to be correct as shown with simulated data [34,35]. Therefore, as 'best practice', we ran analyses using data representing the Neoaves ingroup only (Figure 2) and then included the Galloanserae outgroup [1,2] for comparison (Figure 3). The main conclusion is that neither unconstrained tree has the seven Metaves taxa together. The Metaves question will be discussed first, then the other aspects of the tree later.

We then constrained the seven Metaves taxa (kagu, tropicbird, flamingo, two grebes, hummingbird, and swift) to form a clade (as in Figure 1A). In order to have the same model, the optimal parameters from ModelTest were estimated on both trees. Both the unconstrained MrBayes and ML trees are highly significantly better than the constrained Metaves tree. The likelihood values for the ML trees are -162992.8 and -163611.6 respectively (≈ 620 log-likelihood units difference, and on the Shimodaira-Hasegawa p ≈ 0.0000). Considering that the SH test is regarded as a conservative test [36], the mitochondrial data strongly reject the Metaves/Coronaves subdivision. This leads us to ask why the seventh intron of the β-fibrinogen gene gives support for the Metaves/Coronaves division.

### Seventh intron of the β-fibrinogen gene

Thus far, the seventh intron of the β-fibrinogen gene (FGB-int7) is the only locus giving direct support for the Metaves/Coronaves subdivision. Alternative hypotheses were tested regarding the alignment and the number of copies of this intron. We reduced the number of taxa to analyse the FGB-int7 sequences (see methods), and resolved the Metaves clade using ML and Maximum Parsimony (MP) although monophyly of Coronaves and Metaves had only 54% and 85% bootstrap support, respectively (5000 MP replicates). From a total of 1075 sites (although, as aligned in the full dataset there are 1930 sites) there are 22 parsimony sites for Metaves. Seventeen of these parsimony sites occur in the first 400 positions of the alignment, although there is no obvious clustering (around indels for example). Trees reconstructed following alternative alignments using the addition of simulated data from the mtDNA tree still recovered Metaves as a group. This was also the case when the sequences were reversed and realigned [30].

No paralogous of FGB-int7 were found from the 26 clones that were sequenced from one Metaves (kagu), and one Coronaves (a New Zealand rail, weka, *Gallirallus australis*). Occasionally single nucleotide differences were found between clones from the same bird, but certainly no changes that would indicate duplicate copies that may have diverged 60–80 Mya [2,37]. Thus no evidence of paralogs was found, and so this does not appear to be a likely explanation for the Metaves grouping.

### Avian evolution

An unexpected feature of our analysis of the whole mitochondrial DNA sequences (Figure 3) was that the MrBayes topology (outgroup included) when enforced in PAUP* provided a tree with a lower likelihood score than the best ML tree found using heuristic search (although not significantly different with SH test (p = 0.486)). These two trees are similar; differing in their placement of the flamingo/grebe clade as sister to the shorebirds (Charadriiformes; gull/turnstone/oystercatcher). The heuristic search ML tree put the flamingo/grebe clade between the falcon clade and the rest of the water-carnivores, while the better MrBayes tree puts the flamingo-grebe as sister to shorebirds. We looked at the stability of this clade by reducing noise in the data set using site-stripping. This method systematically RY-codes or removes the most variable nucleotide sites (scored as the average of their consistency and retention indices) and then re-runs MrBayes ([38] and see methods section below). The flamingo/grebe (Mirandornithes) and flamingo/grebe/shorebirds clades were returned in all MrBayes analyses, even when 7.12% of sites were re-coded as RY and a further 10% of sites were excluded. In contrast, the pelican and frigatebird were no longer grouped together at this level of site stripping, though the hummingbird/swift clade was retained. Swift and hummingbird (Apodiforms) came together in all trees, falling outside the large and very diverse group of raptors, shore and sea birds [2].

Kagu and takahe (rail) did not come together in any of our unconstrained trees (Figures 2, 3). The kagu is basal to the passerine/woodpecker/aracari clade and our rail representative (takahe) basal to the hummingbird/swift clade. However, when we constrained kagu and takahe to be monophyletic the resulting tree was not significantly worse than our best tree (SH test; P = 0.181). Some of the uncertainty in our dataset is illustrated on the MrBayes consensus network (Figure 2) – some partitions place the takahe basal to the hummingbird/swift, and some place it outside this clade but still basal to the water-carnivores. Similar uncertainty is seen on the kagu lineage where we have signal for kagu to be basal to the woodpecker/aracari clade. The ability to show the presence of both signals (for example, takahe basal to the water-carnivore clade; or basal to swift/hummingbird; Figure 2) is an important advantage of combining tree and network methods [39].

## Discussion

The phylogenetic hypotheses (Figures 2, 3) generated by our whole mitochondrial genome sequences using different models are generally concordant. The relationships among the Neoaves (the ingroup) differ little whether or not the outgroup (Galloanserae) is included (Figures 2, 3). Where the two phylogenies differ is in small changes in the position of the loon, penguins, albatross/petrel, stork/heron, and pelican/frigatebird. In all other respects the inclusion of the Galloanserae does not affect the relationships resolved and thus the basic stability of the tree is observed.

The seven birds in our mitochondrial sequence dataset that represent elements of the Metaves group do not form a monophyletic clade. Ericson and co-workers [7] also failed to obtain the Metaves clade using four nuclear genes, although they could with the inclusion of FGB-int7. No other dataset, molecular nor morphological, has found direct evidence for the division of the Neoaves into Metaves and Coronaves [8,9,17]. Nor could Fain and Houde [3] identify a single shared derived morphological character to unit the members of Metaves. We cannot explain why FGB-int7 provides a division not seen in any other data set but this division is based on fewer than 25 nucleotide sites (approximately 2% of their data), and the monophyly of these two clades were supported by only 85% and 60% of maximum parsimony bootstraps. It should be noted that Fain and Houde [3] characterize the members of Metaves as taxomonically problematic (p. 2565) and our analyses indicates why this has been the case for both the kagu and tropicbird. From our mitochondrial sequences we can infer that neither kagu nor tropicbird are closely related to other members of the orders into which they are currently placed (Gruiformes and Pelecaniformes, respectively). However, there is no suggestion in our mitochondrial data that we should unite these 'problematic' taxa into a single clade [3].

Given the contradictory evidence from mtDNA, we appraised the quality of phylogenetic signal from FGB-int7 data that returned monophyly of the Metaves clade with weak support. However, we did not find evidence for obvious sources of the anomalous phylogeny; we found no indication that FGB-int7 has a paralog within birds and the alignment appears reasonable. So why does this one gene return a pattern not seen from any other characters? The intron is evolving rapidly enough to accumulate differences between sister species and has been used to resolve phylogenetic relationships within and among genera (e.g. woodpeckers [40]; amphibians [41]). However, the alignment of an intron with many indels and no constant sites could lead to the formation of artifact clades when studying relationships among orders that are more than 60 million years old. We note, again, that with our alignment of mitochondrial proteins, we exclude sites between a gap and a site that is constant across the dataset, and if we applied this conservative alignment criterion, we would have to exclude all FGB-int7 sites. Overall, our conclusion is that the whole mitochondrial genome data strongly disagrees with the Metaves/Coronaves split, but the 22 parsimony sites in the FGB-int7 uniting the Metaves remain unexplained. Given the complete absence of support from other markers we doubt the FGB-int7 pattern is due to the shared evolutionary history of the bird species within Metaves. Nevertheless, we expect that FGB-int7 will be an excellent marker for studying evolution within genera and families where there should be no problems of alignment.

The Metaves/Coronaves split returned from analysis of FGB-int7 provided an intriguing framework in which to explore the evolution of avian ecology. The split, apparently characterized by multiple examples of convergent evolution (independent origins of similar feeding ecology/behavior) indicated a clear phylogenetic and hence historical dichotomy; a major, old division in the evolution of birds. Given the failure of any additional nuclear or mtDNA sequence data to support either of the Metaves or Coronaves clades and the clear contradiction from morphology and current taxonomy (e.g., five orders of birds are rendered polyphyletic by FGB-int7), where does this leave observations of convergent evolution in birds? Clearly examples of convergence of form and behavior exist across the phylogeny of birds and were well recognized prior to the proposal of the Metaves/Coronaves split. Of the three more controversial examples of avian ecological convergence proposed by Fain and Houde [3] we can comment on two: tropicbirds and boobies as pelagic soaring plunge divers, and grebes and loons as "foot-propelled divers with high wing loading".

Two of the three Pelecaniformes represented are sister taxa; frigatebird and pelican (Figures 2, 3), in agreement with Livezey and Zusi [9]. But in contrast to Livezey and Zusi [9], the tropicbird does not appear to belong to this clade. Placement of the topicbird is still difficult because trees of almost equal value place it basal to raptors (buzzard/osprey/eagle MrBayes; or falcons ML), but never with other members of Metaves. Our findings support those from DNA-DNA hybridization where tropicbirds were basal to a large clade of aquatic birds that includes the New World Vultures [22]. The tropicbird is not part of the Pelecaniform clade (represented here by the pelican and frigatebird) which also includes the boobies [42], and therefore the similar feeding methods of the tropicbird and boobie look likely to have resulted from convergent evolution. Similarly, although the grebes and loons are part of the same large clade they are not sister taxa and so their similar appearence and diving methods must also constitute convergence. In conjunction with the nine other examples of avian morphological convergent evolution [3] one can conclude that the process is not an uncommon phenomenon within the bird radiation in general. However, these examples of morphological and ecological convergence do not support the Metaves/Coronaves division.

We find support in the whole mitochondrial genome data set for the sister relationship of swifts and hummingbirds (Apodiformes), and find support for the grouping of flamingo and grebe (the Mirandornithes of Sangster [19]). This may well form a sister clade to the Charadriiformes (shorebirds); our site-stripping technique showed this relationship to be stable, from which we infer a common evolutionary history. In keeping with this idea, morphological studies of extant and fossil species reveal that early flamingos resembled more typical wading birds and place the Phoenicopteridae within the order Charadriiformes [16].

We find little support for the hypothesis that the kagu is related to the rails, although when forced together the resulting tree was not significantly worse. Kagu appears to have no close allies in our data set and has long been recognized as a difficult species to place [12]. Cracraft [11] and others place kagu and rails within the Gruiformes along with the mesites, bustards, seriemas, sunbittern, sungrebe, trumpeters and cranes. In contrast, Livezey and Zusi [9] found the rails to be sister to the shorebirds (Charadriiformes), and the Gruiformes (including kagu) to be sister to this pair. As the takahe and kagu are (unexpectedly) not allies we now have two long edges (branches) within our phylogeny that have the potential to disrupt stable clades. Addition of sunbittern to break-up the kagu long edge, and a crane, or another rail to break the takahe long-branch should solve this problem.

## Conclusion

We conclude that avian mitochondrial genomes reject the hypothesis of a shared evolutionary history for hummingbirds, kagu, tropicbirds and flamingos. Although some major Neoaves clades remain to be sequenced (for example cuckoos and pigeons) it is very unlikely that the addition of any avian species could force the seven Metaves species in this dataset into a monophyletic clade. The phylogenetic tree of 41 bird species represented here has provided new hypotheses such as the sister relationship of the shorebirds and the flamingo/grebe clade that can now be tested with other datasets.

## Methods

### The birds

The ruby-throated hummingbird (*Archilocus colubris*) tissue was provided by the Louisiana State University Museum of Natural Science and is sample LSUMNZ B-26279. The Australian little grebe (*Tachybaptus novaehollandiae*) is from the Australian Museum Sydney (sample EBU 9986). The New Zealand rail (takahe: *Porphyrio hochstetteri*) was provided by the Department of Conservation via Massey University Veterinary Pathology, and the New Caledonian kagu (*Rhynochetos jubatus*) sample was a gift from Christophe Lambert, New Caledonia. The common swift (*Apus apus *AM237310 EMBL) was provided by Stefan Gabrielsson (Katastrofhjälp fåglar och vilt, Kristianstad/Bromölla) and the greater flamingo (*Phoenicopterus ruber roseus*) came from the Auckland Zoological Park.

Genomic DNA was extracted from the hummingbird, kagu, rail, flamingo and grebe tissue at the AWC using 25–50 mg of liver and the High Pure™ PCR Template Preparation Kit (Protocol Vb; Boehringer Mannheim) according to the manufacturers instructions. To minimize the possibility of obtaining nuclear copies of mitochondrial genes (numts), mitochondrial genomes were first amplified in 2–3 long overlapping fragments (3.5 – 12 kb in length) using the Expand ™ Long template PCR System (Roche). The products were excised from agarose gel using Eppendorf gel extraction columns. Long-range PCR products were then used as templates for multiple rounds of short-range PCR of overlapping fragments 0.5 – 3 kb in length. Primers were found from a database maintained in our laboratory and described by Slack et al. [43]. Sequencing was performed using BigDye^® ^Terminator Cycle Sequencing reagents according to the manufacturers instructions (Applied Biosystems), and the nucleotide sequences read on an ABI 3730 automated sequencer (Applied Biosystems). For each genome, overlapping sequence fragments were assembled and checked for ambiguity using Sequencher™ 4.2.2 (Gene Codes Corp.).

Where necessary PCR products were cloned using standard techniques to resolve length heteroplasmy in control regions arising from microsatellite repeats [1]. At least three clones were sequenced for each region to guard against PCR errors. In all cases, overlaps between sequences were sufficient to ensure synonymy (usually ≥ 100 bp between sequences from short-range PCR; and a total of 1 – 4 kb between the different long-range products. Sequence identity was confirmed through BLAST searches of the NCBI database [44], confirmation of amino acid translation in coding regions and alignment with other species.

In addition to the six new bird mitochondrial genomes reported in this paper, 35 other complete avian mt genomes were included in the analyses, 29 neoaves and six Galloanserae. The Galloanserae taxa are: chicken (*Gallus gallus*; GenBank accession number AP003317), Japanese quail (*Coturnix japonica*; AP003195), magpie goose (*Anseranas semipalmata*; AY309455), redhead duck (*Aythya americana*; AF090337), greater white-fronted goose(*Anser albifrons*; AF363031), Australian brush-turkey (*Alectura lathami*, AY346091). The 29 neoaves taxa are: rifleman (NZ wren, *Acanthisitta chloris*; AY325307), gray-headed broadbill (*Smithornis sharpei*; AF090340), fuscous flycatcher (*Cnemotriccus fuscatus*; AY596278), superb lyrebird (*Menura novaehollandiae*; AY542313), village indigobird (*Vidua chalybeata*; AF090341), rook (*Corvus frugilegus*; Y18522), ivory billed aracari (*Pteroglossus azara*, DQ780882), woodpecker (*Dryocopus pileatus*; DQ780879), peregrine falcon (*Falco peregrinus*; AF090338), forest falcon (*Micrastur gilvicollis*, DQ780881), American kestral (*Falco sparverius*, DQ780880), Eurasian buzzard (*Buteo buteo*; AF380305), osprey (*Pandion haliaetus*, DQ780884), Blyth's hawk eagle (*Spizaetus alboniger*, AP008239), turkey vulture (*Cathartes aura*, AY463690), blackish oystercatcher (*Haematopus ater*; AY074886), ruddy turnstone (*Arenaria interpres*; AY074885), southern black-backed gull (*Larus dominicanus*, AY293619), Oriental stork (*Ciconia boyciana*; AB026193), red-throated loon(*Gavia stellata*; AY293618), little blue penguin(*Eudyptula minor*; AF362763), black-browed albatross(*Diomedea melanophris*; AY158677) and Kerguelen petrel (*Pterodroma brevirostris*; AY158678), white-faced heron (*Ardea novaehollandiae*; DQ780878), rockhopper penguin (*Eudyptes chrysocome*; NC 008138), great crested grebe (*Podiceps cristatus*; NC 008140), frigatebird (*Fregata sp*; AP009192), Australian pelican (*Pelecanus conspicillatus*, DQ780883), red-tailed tropicbird (*Phaethon rubricauda*; AP009043). Paleognath taxa were not included because the paleo/neognath division has been well established for mitochondrial genomes[1,2]. Thus we rooted our Neoaves trees with the six Galloanserae sequences.

### Phylogenetic Analysis

Nucleotide sequences for each gene were aligned separately in Se-Al v2 [45]. Protein-coding genes were aligned using translated amino acid sequences and RNA genes were aligned based on secondary structure. The resulting dataset has 12 protein-coding genes, two rRNA genes and 21 tRNAs (lacking tRNA-Phe because sequence data is missing in some taxa). Gaps, ambiguous sites adjacent to gaps, the NADH6 (light-strand encoded), and stop codons (often incomplete in the DNA sequence), were excluded from the alignment. The full analysed mtDNA dataset was 13,229 bp in length.

In previous work [46-48] we found that RY-coding of the most variable partitions of the nucleotide data (especially the 3^rd ^codon position) was advantageous. This recoding increases the proportion of changes on internal branches of the tree (that is, 'treeness'), reduces effective differences in nucleotide composition (relative compositional variability; RCV), and was shown to increase concordance between mitochondrial and nuclear datasets. RY-coding does improve the ML scores, but because RY-coding is not strictly nested within nucleotide-coding (M.A. Steel, pers. comm.) it is not valid to compare their respective ML scores directly. However, because of the better fit of the data to the model (higher treeness, and lower RCV) this has been our preferred method of analysis of vertebrate mitochondrial data. Thus the trees reported here have the third codon positions of 12 protein-coding genes recoded as R (instead of A & G), and Y (instead of C & T). The full data set is available [49]. Analysis used standard programs including ModelTest [50] PAUP*4.0b10 [51], MrBayes 3.1.2 [52], and consensus networks [39]. We ran 1000 unconstrained ML bootstrap replicates with PAUP*4.0b10 on the Helix computing cluster [53], plus a Bayesian analysis using chains of 10^7 ^generations. For some runs, we constrained the seven 'Metaves' taxa to be monophyletic (see Figure 1) and used a Shimodaira-Hasegawa (SH) test [54] implemented in PAUP* to compare this ML tree with the unconstrained ML tree (RELL, one-tailed test, 1000 bootstrap replicates).

### Site-stripping

The most serious problem for reconstructing deep-level phylogeny from mitochondrial sequences is substitution saturation [55,56]. Aside from the direct effect of superimposed substitutions eroding phylogenetic signal, 'non-historical' biases (such as that derived from compositional non-stationarity) accumulate more rapidly at faster evolving sites. In a number of recent studies [56,57] we have attempted to reduce these problems by identifying partitions among which the sites have (on average) high signal erosion and then either RY-code them (using only information from the slower transversions), or excluding that partition altogether. This earlier approach may not be optimal as some phylogenetically useful sites are excluded simply because they group under some prior definition (e.g. codon position) with many fast evolving sites. So here we test a noise reduction technique in which the information retained from the sequence is determined on a site-by-site basis. 'Noise reduction', in general, is a standard technique in many areas of science [58].

In an earlier noise reduction technique using site-stripping [38], sites were excluded from analysis if changes occurred at these sites within a few predefined closely-related taxa. As a proxy for their utility, in the present study, sites are scored as the average of their consistency and retention indices (CI and RI, respectively). The CI and RI are calculated on the consensus tree that upholds relationships among the primary data matrix that are uncontroversial with respect to prior studies, and also receive a Bayesian posterior probability of 1.00 in the unstripped analyses. Any groupings that do not conform to these requirements are collapsed so as to avoid the circularity of increased support resulting from the exclusion of sites that might have been influenced by conflict with that grouping. The consensus tree we used was: ((((quail, chicken), brush turkey), ((goose, duck), magpie goose)),((((rook, indigobird), lyrebird), (broadbill, flycatcher), rifleman),((gull, turnstone), oystercatcher), flamingo,(great crested grebe, grebe), takahe, hummingbird, kagu, heron, (petrel, albatross), (little blue penguin, rock hopper penguin), stork, turkey vulture, tropicbird, ((falcon, kestrel), forest falcon), ((hawk eagle, buzzard), osprey), swift, pelican, frigatebird, (aracari, woodpecker), loon)).

Variants of the primary dataset were RY-coded and site-stripped at progressively higher threshold levels of site utility, (CI+RI)/2 = 0.08, 0.12, 0.16, 0.20, 0.24, using the Perl program site_strip_search.pl [49] For each iteration, individual site utility scores that fall below specified threshold levels are RY-coded. When the resulting site utility score remains below the specified level, then the site is excluded altogether. Bayesian inference analyses were carried out on each of these 'noise reduced' data matrices.

### The seventh intron of the β-fibrinogen gene

Fain and Houde's [3] dataset of the seventh intron of the β-fibrinogen gene (FGB-int7) was reduced to 35 sequences, corresponding to the 35 taxa common with our mitochondrial dataset [see Additional file 1]. The 35 taxa include pairs that represent equivalent branching patterns (the same position in a cladogram relative to the other taxa in each dataset) although the species are not always identical, or in some cases, even sister taxa. The alignment of the taxon-reduced FGB-int7 dataset was checked visually in Se-Al v2, with the Metaves group at the top, and the dataset exported as a Mega file. In this format, the positions of the sites that potentially contribute to phylogenetic signal in the dataset, could be examined and compared. To evaluate the utility of the intron positions, phylogenetic analyses were conducted by equally weighted maximum parsimony (MP), with indel characters treated as missing data using PAUP* 4.0b10 [51].

We tested whether tree reconstruction from the 35-taxa FGB-int7 data is stable with respect to the internal reference tree generated during the Clustal X alignment. A 500 nucleotide dataset was simulated in Seqgen 1.3 [59] on the mitochondrial tree, under the ML-GTR+I+Γ optimisation for the original mt data. Only the 35 taxa common to both the FGB-int7 and mitochondrial datasets were included in the simulated tree. Insertion of the simulated data ahead of the FGB-int7 sequences should drive the alignment to conform more closely to the original mt tree. We then performed phylogenetic analyses using the combined alignment (simulated sequences plus FGB-int7 sequences) and using only the realigned FGB-int7 sequences.

In addition, we reversed the sequences of the FGB-int7 dataset, and re-aligned it using Clustal X as recommended by Landan and Graur [30]. This reversed dataset was also used for phylogenetic analyses. Landan and Graur [30] have shown that reversing the direction of the sequences before an alignment is made can result in quite different trees from the unreversed alignment if the true alignment is ambiguous. The change in result arises because many programs, when faced with tied values may always take the first alternative (i.e., not breaking ties randomly). Conversely, an alignment that is robust to reversing the sequences supports the original alignment. Finally, using the primers FIB-BI7U and FIB-BI7L [60] we amplified the FGB-int7 from two birds; one Metaves (kagu), and one Coronaves (a New Zealand rail, weka, *Gallirallus australis*). The products were cloned and a total of 26 clones were sequenced to detect possible paralogous copies in the same genome. Thus we had a range of approaches to test the robustness of the *β*-fibrinogen seventh intron data.

## Authors' contributions

MMR sequenced the flamingo and hummingbird mtDNA, preformed phylogenetic analyses and worked on the manuscript. SAT sequenced the takahe mtDNA and participated in the writing. ABH sequenced the swift mtDNA. OK sequenced the kagu and grebe mtDNA. MJP participated in the phylogenetic analysis and developed the site-stripping proceedure, PAM participated in the sequence alignments, cloning and analysis of the FGB-int7 sequences. DP participated in the design of the study and analyses of the data, obtained funding and helped draft the manuscript. All authors read and approved the final manuscript.

## Supplementary Material

Additional file 1 Birds with both mitochondrial and nuclear intron sequence data. The 35 birds common (or phylogenetically equivalent) to both mitochondrial (this paper) and the seventh intron of the β-fibrinogen datasets (Fain and Houde 2004), used to explore the phylogenetic signal within the seventh intron of the β-fibrinogen sequence. Click here for file
