# Supplementary material for: Bird evolution: testing the Metaves clade with six new mitochondrial genomes
Source: BMC Evol Biol. 2008 Jan 23;8:20. doi: 10.1186/1471-2148-8-20 (PMC2259304; doi:10.1186/1471-2148-8-20)
Supplement: Additional file 1 — Birds with both mitochondrial and nuclear intron sequence data. The 35 birds common (or phylogenetically equivalent) to both mitochondrial (this paper) and the seventh intron of the β-fibrinogen datasets (Fain and Houde 2004), used to explore the phylogenetic signal within the seventh intron of the β-fibrinogen sequence. [file 1471-2148-8-20-S1.pdf]

Mitochondrial		7th Intron β-fibrinogen
Aythya americana	duck	*Aythya collaris*
Anser albifrons	goose/whistling duck	*Dendrocygna bicolor*
Gallus gallus	chicken	*Gallus gallus*
Alectura lathami	brush-turkey/scrub fowl	*Megapodius reinwardt*
Cnemotriccus fuscatus	New World flycatcher	*Contopus virens*
Vidua chalybeata	indigobird	*Catharus guttatus*
Corvus frugilegus	rook/blue jay	*Cyanocitta cristata*
Pteroglossus azara	aracari	*Pteroglossus azara*
Dryocopus pileatus	woodpecker	*Picoides sp*
Rhynochetos jubatus	kagu	*Rhynochetos jubata*
Haematopus ater	oystercatcher	*Haematopus ostralegus*
Arenaria interpres	turnstone	*Tringa melanoleuca*
Larus dominicanus	gull	*Larus occidentalis*
Phoenicopterus ruber roseus	flamingo	*Phoenicopterus ruber*
Tachybaptus novaehollandiae	grebe	*Podilymbus podiceps*
Podiceps cristatus	grebe	*Aechmophorus clarkii*
Ciconia boyciana	stork	*Ciconia maguari*
Ardea novaehollandiae	heron	*Egretta tricolor*
Pelecanus conspicillatus	pelican	*Pelecanus erythrorhynchus*
Fregata sp.	frigatebird	*Fregata minor*
Gavia stellata	loon	*Gavia immer*
Eudyptula minor	penguin	*Eudyptyla minor*
Diomedea melanophris	albatross/shearwater	*Puffinus tenuirostris*
Pterodroma brevirostris	petrel	*Oceanodroma leucorhoa*
Cathartes aura	turkey vulture	*Cathartes aura*
Pandion haliaetus	osprey	*Pandion haliaetus*
Buteo buteo	buzzard	*Buteo jaimaicensis*
Phaethon rubricauda	tropicbird	*Phaethon aethereus*
Micrastur gilvicollis	forest-falcon/mousebird	*Colius striatus*
Falco peregrinus	falcon	*Polihierax semitorquatus*
Falco sparveerius	kestrel	*Falco sparverius*
Porphyrio hochstetteri	takahe/swamp hen	*Porphyrio porphyrio*
Archilocus colubris	hummingbird	*Amazilia tobaci*
Apus apus	swift	*Aerodramus salanganus*
Anseranas semipalmata	magpie goose	*Anseranas semipalmata*
